# MutS HOMOLOG1 silencing mediates *ORF220* substoichiometric shifting and causes male sterility in *Brassica juncea*

**DOI:** 10.1093/jxb/erv480

**Published:** 2015-10-29

**Authors:** Na Zhao, Xinyue Xu, Yashitola Wamboldt, Sally A. Mackenzie, Xiaodong Yang, Zhongyuan Hu, Jinghua Yang, Mingfang Zhang

**Affiliations:** ^1^Laboratory of Germplasm Innovation and Molecular Breeding, Institute of Vegetable Science, Zhejiang University, Hangzhou 310058, China.; ^2^Department of Agronomy and Horticulture, University of Nebraska, Lincoln, NE 68588-0660, USA.; ^3^Key laboratory of Horticultural Plant Growth, Development & Quality Improvement, Ministry of Agriculture, Hangzhou 310058, China.; ^4^Zhejiang Provincial Key Laboratory of Horticultural Plant Integrative Biology, Hangzhou 310058, China.

**Keywords:** Cytoplasmic male sterility, DNA recombination, mitochondrial DNA, MSH1, pollen, substoichiometric shifting.

## Abstract

Cytoplasmic male sterility (CMS) has consistently been associated with the expression of mitochondrial open reading frames (ORFs) that arise from genomic rearrangements. Spontaneous fertility reversion in CMS has been observed in several cases, but a clear understanding of fertility reversion controlled by nuclear genetic influences has been lacking. Here, we identified spontaneous fertile revertant lines for *Brassica juncea* CMS cytoplasm in which the mitochondrial genome has undergone substoichiometric shifting (SSS) to suppress *ORF220* copy number. We placed ORF220, with or without a mitochondrial targeting presequence, under the control of the *CaMV35S* and *AP3* promoters in Arabidopsis to confirm that *ORF220* causes male sterility when mitochondrially localized. We found that copy number of the *ORF220* gene was altered under conditions that suppress MSH1, a nuclear gene that controls illegitimate recombination in plant mitochondria. *MSH1*-*RNAi* lines with increased *ORF220* copy number were male sterile compared with wild type. We found that a wide range of genes involved in anther development were up- and down-regulated in revertant and *MSH1-RNAi* lines, respectively. The system that we have developed offers valuable future insight into the interplay of MSH1 and SSS in CMS induction and fertility reversion as a mediator of nuclear–mitochondrial crosstalk.

## Highlight

*MSH1* participates in fertility reversion and male sterility by mediating mitochondrial genomic substoichiometric shifting of *ORF220* and altering expression of anther development-associated genes in *Brassica juncea*.

## Introduction

Cytoplasmic male sterility (CMS) is a maternally inherited trait that prevents the production of functional pollen. The phenomenon, observed in >150 plant species with several conserved features in common, comprises one of very few systems of nuclear–mitochondrial genetic interaction amenable to study in higher plants. CMS has been associated with expression of novel mitochondrial open reading frames (ORFs) that arise by rearrangement of mitochondrial genomes (Hanson and Bentolila, 2004; Woodson and Chory, 2008). Fertility restorer (Rf) genes—pentatricopeptide repeat (PPR) proteins in most cases—are examples of nuclear genes that can alter mitochondrial CMS-associated gene expression. The PPR proteins usually operate at post-transcriptional levels, by RNA editing, processing, and polyadenylation, as well as post-translationally (Hanson and Bentolila, 2004; Schmitz-Linneweber and Small, 2008). In the system of Wild Abortive CMS in rice, for example, restorer genes *Rf4* and *Rf3* suppress the CMS-associated WA352 gene at transcriptional and translational levels, respectively (Luo *et al.*, 2013).

Spontaneous fertility reversion in CMS has been seen in several plant species, and serves as an alternative means in nature to overcome mitochondrially encoded male sterility (Bellaoui *et al.*, 1998; Arrieta-Montiel *et al.*, 2001). Spontaneous fertility reversion in CMS is generally characterized by mitochondrial genomic substoichiometric shifting (SSS) (Arrieta-Montiel and Mackenzie, 2010), with the frequency of these genomic changes influenced by nuclear genetic background (Mackenzie *et al.*, 1988; Small *et al.*, 1988). An individual nuclear gene has been shown to reproducibly direct particular mitochondrial rearrangement events in common bean (Mackenzie and Chase, 1990), and natural or induced nuclear gene mutation can cause fertility reversion in carrot (Chahal *et al.*, 1998) and rice (Shen *et al.*, 1996). Tissue culture conditions can also give rise to fertility reversion in petunia and maize, again in association with mitochondrial genomic changes (Clark *et al.*, 1988; Small *et al.*, 1988; Fauron *et al.*, 1990). However, the nuclear genes controlling mitochondrial genomic recombination to effect fertility reversion have not been identified in most cases.

The plant mitochondrial genome is known to undergo high frequency recombination and to comprise a multipartite organization (Arrieta-Montiel and Mackenzie, 2010; Marechal and Brisson, 2010). Asymmetric DNA exchange at small repeats appears to influence the stoichiometry of subgenomic mtDNA molecules—a phenomenon termed SSS (Small *et al.*, 1989). This recombination is influenced by nuclear genes, including RecA3 and MSH1, which suppress ectopic mitochondrial recombination (Abdelnoor *et al.*, 2003; Shedge *et al.*, 2007). In Arabidopsis *msh1*, over 47 recombination repeat pairs become differentially active in the mitochondrial genome (Davila *et al.*, 2011). Disruption of *MSH1* has been shown to result not only in mitochondrial SSS, but also the appearance of CMS in several crops (Sandhu *et al.*, 2007). Mitochondrial genome recombination plays an important role in plant mitochondrial genome evolution (Small *et al.*, 1989; Chang *et al.*, 2011b), generating novel mitotypes (Chen *et al.*, 2011), and environmental adaptation (Shedge *et al.*, 2010; Xu *et al.*, 2011).

We previously developed a CMS line of *Brassica juncea* and identified the CMS-associated locus *ORF220* in the mitochondrial genome (Yang *et al.*, 2010). In this study, we identified fertile revertant lines in CMS *B. juncea*. We then established a link between *MSH1* and mitochondrial genome rearrangements, effecting *ORF220* SSS in association with fertility reversion. We suggest that the MSH1–mitochondrial interaction in plants may participate in the reversible male sterility–fertility transitions involved in gynodioecious reproductive systems.

## Materials and methods

### Plant materials

CMS and its fertile maintainer lines of *B. juncea* were used for identification of revertant lines and development of *MSH1-RNAi* lines. Wild type (WT) Arabidopsis (*Arabidopsis thaliana*) (Col-0) was used for transformation of *ORF220* with and without a mitochondrial-targeting peptide under 35S (CaMV 35S) and AP3 (APETALA3) promoters. The mitochondrial targeting peptide was amplified from a previous construction plasmid (He *et al.*, 1996). WT Arabidopsis (Col-0) was used for amplification of AP3 promoter sequences. A fertile isogenic maintainer line of *B. juncea* was used to generate the *MSH1-RNAi* line.

### Mitochondrial genome assembly

Total DNA was isolated from fresh leaves of CMS and REV19 lines using a cetyl trimethylammonium bromide (CTAB) protocol. Total genomic DNA was prepared in paired-end libraries, tagged and sequenced on the Illumina Hiseq2500 platform. High quality reads were mapped to the *B*. *juncea* mitochondrial genome sequence (Genbank: KJ461445) using SAOP2, and paired mapping reads were extracted for mitochondrial genome assembly. These reads were assembled into scaffolds with the Velvet program (Zerbino and Birney, 2008).

### DNA gel blotting and SSS of ORF220

Total genomic DNA samples were extracted from leaves for DNA gel blotting and SSS analysis of *ORF220*. For blotting, total genomic DNA samples were digested with *Hind*III endonuclease (Takara, Japan). Digested DNA samples were separated by electrophoresis for 24h, and were transferred and fixed to HyBond N^+^ nylon membrane (Amersham, Sweden) by capillary method. The *ORF220* probe was prepared by PCR with the DIG probe synthesis kit (Roche, Switzerland). Hybridization was performed by standard pre-hybridization, probe denaturation, and hybridization in solution. The hybridization signal was detected using DIG High Prime DNA Labeling and Detection Starter II kit (Roche, Switzerland).

SSS of *ORF220* was monitored by varying PCR amplification cycles. The PCR reaction was performed in a total volume of 50 μl containing 5 μl 10× Ex Taq Buffer (Mg^2+^ plus), 4 μl 10mM dNTP, 10 pmol of forward and reverse primers, 200ng of template DNA and 0.25 μl Ex Taq^TM^ DNA Polymerase. The amount of template DNA was adjusted to be equal in each sample. The PCR solutions were incubated for 5min at 94 °C, and then run for 25, 30, and 35 cycles, respectively, at 94 °C for 30s, 50 °C for 30s, and 72 °C for 60 s, followed by final extension at 72 °C for 10min. The amplifications were separated by electrophoresis on 1% agarose gels. The primers used for *ORF220* SSS assays are listed in Supplementary Table S1 at *JXB* online.

### Expression analysis of ORF220

RT-PCR and real-time RT-PCR were used for transcriptional expression of *ORF220*. Protein gel blotting was employed to investigate translational expression of ORF220. ORF220 polyclonal antibodies were prepared by BGI Protein (BGI, China). Plant total proteins were extracted from floral buds using Plant Protein Extraction kit (BestBio, China). Plant proteins were separated in 5% and 12% gradient acrylamide gels, and were transferred to PVDF membrane and immunoblotted with anti-ORF220 polyclonal antibodies. The membrane was then combined with Enhanced Luminol Reagent and Oxidizing Reagent substrates. The signals were detected by FUJIFILM LAS-300 Luminescent Image Analyzer (FUJIFILM, Japan). The primers used for *ORF220* assay are listed in Supplementary Table S1 at *JXB* online.

### ORF220 construction and transformation in Arabidopsis

cDNA of *ORF220* and the mitochondrial presequence were amplified according to our previous publication (Yang *et al.*, 2010). The ORF220 and mt-ORF220 constructions were inserted into pMDC83 binary expression vector to generate *ORF220*-pMDC83 by gateway protocols (Curtis and Grossniklaus, 2003). Four constructions of *ORF220* plasmid were introduced into *Agrobacterium tumefaciens* strain (GV1301) and the floral dipping method was used to introduce the *ORF220* construction to Arabidopsis (Clough and Bent, 1998). PCR and RT-PCR amplifications of *ORF220* confirmed successful transformations. WT and transgenic Arabidopsis T_2_ lines were used in this study. The primers used for *ORF220* constructions are listed in Supplementary Table S1 at *JXB* online.

### *MSH1-RNAi* line construction

The *MSH1-RNAi* construction was prepared identically with that reported in tomato previously (Sandhu *et al.*, 2007). The *MSH1-RNAi* construct was made by cloning part of *MSH1* (AT3G24320) domain VI in the RNAi vector pFGC1008 (Kerschen *et al.*, 2004) using primers MSH1RNAI-AscI-F: AGTCGGCGCGCCATTGAGCCTGAAGCAATAGAATGTC; MSH1RNAI-*Swa*I-R: AGTCATTTAAATGAGGACGTTCC GAAATTACGGTGC; MSH1RNAI-*Spe*I-F: AGTCACTAGTATT GAGCCTGAAGCAATAGAATGTC; MSH1RNAI-*Bam*HI-R: AG TCGGATCCGAGGACGTTCCGAAATTACGGTGC. Presence of MSH1 inserts and correct orientation was confirmed by PCR as well as sequencing using primers MSH1RNAI-AscI-F with *Gus*-5out: AGAGGTTAAAGCCGACAGCA for the left fragment and MSH1RNAI-*Spe*I-F with *Gus*-3out: AAGCAACGCGTAAACTCGAC for the right fragment. The *MSH1-RNAi* plasmid was then introduced into *Agrobacterium tumefaciens* strain GV1301. The transformation procedure generating the *MSH1-RNAi* line of *B. juncea* was as described previously (Yang *et al.*, 2010). Transgenic lines were identified by Hygromycin B selection and by PCR amplification of a junction fragment consisting of vector and MSH1 gene sequence. The primers used for *MSH1-RNAi* line confirmation are listed in Supplementary Table S1 at *JXB* online.

### Transcriptome analysis in CMS and revertant lines of *B. juncea*

Total RNAs isolated from floral buds of CMS and REV19 lines of *B. juncea* were used for global transcript analysis by RNA-seq. The protocols for library construction and sequencing were the standard procedures provided by Illumina (NEBNextUltra^TM^ RNA library Prep kit, Illumina, USA). The sequencing was performed using the Illumina HiSeq™ 2500 System according to the manufacturer’s protocol (50bp single read module). An average of 8.5 G clean reads for each library was used for differential gene expression analysis. For each sequenced library, the read counts were adjusted using the edger program package through one scaling normalized factor. Differential expression analysis was performed using the DEGseq R package. P values were adjusted using the Benjamini & Hochberg method. Corrected P-value of 0.005 and log_2_ (fold change) of 1 were set as the threshold for significantly differential expression. Gene Ontology (GO) enrichment analysis of differentially expressed genes was implemented by GOseq R package, in which gene length bias was corrected. GO terms with corrected P-value <0.05 were considered significantly enriched by differentially expressed genes. Clusters of orthologous groups (COG) analysis was used as an online service (www.ncbi.nlm.nih.gov/COG/).

Differentially expressed genes by RNA-seq were annotated based on whole genome sequence information. Then we selected 15 annotated anther development-associated genes to represent candidate genes involved in Arabidopsis anther development (Chang *et al.*, 2011a). Quantitative (q)PCR was used to study expression patterns of these selected anther development-associated genes. The primers for these anther-related genes are listed in Supplementary Table S1 at *JXB* online.

## Results

### Identification of fertility reversion in *B. juncea*

**Fig. 1.**
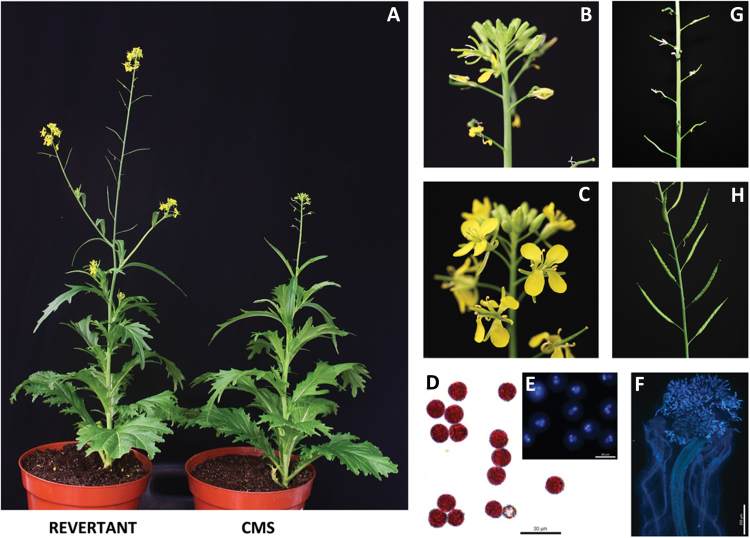
Phenotypes of cytoplasmic male sterile and revertant lines of *B. juncea*. (A) CMS and REV19 plants. (B) CMS inflorescence. (C) REV19 inflorescence. (D) Alexander staining of pollen from REV19. (E) DAPI staining of pollen from REV19. (F) *In situ* germination on stigma of pollen from REV19. (G) Silique from CMS line by self-crossing. (H) Silique of REV19 by self-crossing. (This figure is available in color at *JXB* online).

Fifty-three seeds from self-pollination were collected from 39 CMS *B. juncea* (T84-66A) plants, of which two seeds gave rise to male fertile plants, designated revertants REV19 and REV21, and the remaining were male sterile plants (see Supplementary Table S2 and Supplementary Fig. S1 at *JXB* online). REV19 displayed earlier flowering than the CMS isoline (Fig. 1A), with full flower structure and normal stamens (Fig. 1B, C). Pollen from REV19 appeared normal based on Alexander staining (Fig. 1D), DAPI staining (Fig. 1E), and *in situ* germination on stigmas (Fig. 1F). Consequently, seed set was fully recovered in REV19 compared with the CMS line (Fig. 1G, H). REV19 progeny showed full fertility in three consecutive self-crossed generations, but could not restore fertility to the CMS line in crossing as a pollen parent, indicating that the reversion represents a cytoplasmic event.

### Mitochondrial genome rearrangement and SSS of ORF220 in CMS and REV lines

**Fig. 2.**
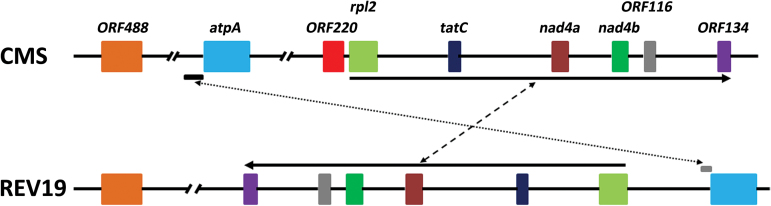
Schematic diagram of *ORF220* and its flanking regions. Dashed lines show rearrangement events. Solid lines represent recombination direction. (This figure is available in color at *JXB* online).

We compared mitochondrial DNA in CMS and REV19 using assembled mt genome scaffolds, confirming that REV19 is not a fertile maintainer line contaminant (see Supplementary Fig. S2 at *JXB* online). We previously identified the CMS-associated *ORF220* from CMS *B. juncea* (Yang *et al.*, 2010). We compared mitochondrial DNA intervals encompassing CMS-associated *ORF220* and flanking regions in the two lines, and observed two genome rearrangements around *ORF220*—a genomic insertion of *atpA* and a reverse complement sequence composed of several mitochondrial genes (Fig. 2). Results indicated that *ORF220* and its flanking regions undergo extensive genomic rearrangement between CMS and REV19 (Supplementary Fig. S3). We also observed several additional mitochondrial rearrangements in other regions of the mitochondrial genomes between CMS and REV19 (Supplementary data).

**Fig. 3.**
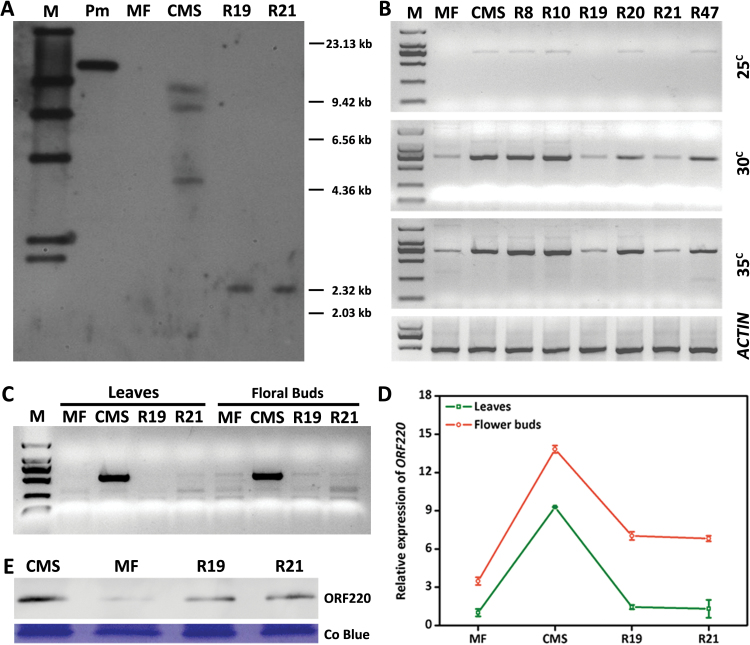
SSS and expression of *ORF220*. (A) DNA gel blot probed with *ORF220* following *Hind*III digestion. Pm (plasmid). (B) SSS analysis of *ORF220*. (C) Steady state transcript levels of *ORF220* by semi-RT-PCR and qRT-PCR (D). (E) Protein levels of ORF220 by protein gel blot analysis. (This figure is available in color at *JXB* online).

Different configurations of CMS-associated *ORF220* were found in the CMS, REV19, REV21, and fertility maintainer (MF) lines, indicated by DNA gel blotting (Fig. 3A). We checked *ORF220* copy number by PCR-based amplification, showing evidence of SSS in the various lines (Fig. 3B). Expression of *ORF220* was significantly increased in the CMS line and decreased in REV19 based on RT-PCR and qRT-PCR (Fig. 3C, D), as well as protein gel blotting (Fig. 3E). The apparent correspondence of ORF220 copy number with gene expression levels in the male sterile and revertant lines suggests that SSS of *ORF220* is associated with fertility reversion in CMS *B. juncea*.

### Mitochondrially targeted ORF220 causes male sterility in Arabidopsis

**Fig. 4.**
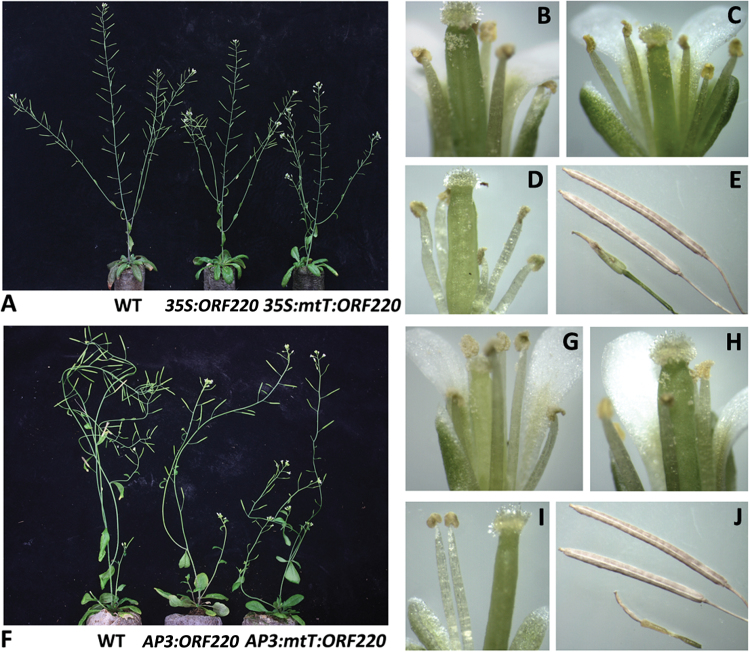
Mitochondrially targeted expression of *ORF220* in *Arabidopsis thaliana*. (A) Transgenic plants with or without mitochondrial (mtT) presequence under control of the CaMV 35S promoter. (B) WT flower. (C) *35S::ORF220* flower. (D) *35S::mtT::ORF220* flower. (E) Silique from WT, *35S::ORF220*, and *35S::mtT::ORF220* plant top-down. (F) Transgenic plants with or without mitochondrial presequence under the AP3 promoter. (G) WT flower. (H) *AP3::ORF220* flower. (I) *AP3:mtT::ORF220* flower. (J) Silique from WT, *AP3::ORF220*, and *AP3::mtT::ORF220* plant top-down. (This figure is available in color at *JXB* online).

To further test the association of ORF220 with male sterility, we developed Arabidopsis lines containing *ORF220* gene constructions with and without a mitochondrial targeting presequence and under control of the CaMV 35S (constitutive) and AP3 (flower-specific) promoters (see Supplementary Fig. S4 at *JXB* online). Plants containing the construct with the 35S promoter, with and without presequence, showed evidence of slightly reduced growth (Fig. 4A). In total, 17 plants that were transformed with the mitochondrially targeting construct were male sterile and plants transformed with the construct lacking presequence showed no evidence of sterility when expressed under control of the 35S promoter (Supplementary Table S3; Fig. 4B–E). Moreover, in constructs containing the AP3 promoter, 28 plants containing the construct with mitochondrial presequence were male sterile and one plant with the construct lacking presequence showed male sterility (Supplementary Table S3; Fig. 4G–J). These results are consistent with our hypothesis that mitochondrially localized *ORF220* causes male sterility.

### Phenotypes and ORF220 SSS in *MSH1-RNAi* lines

**Fig. 5.**
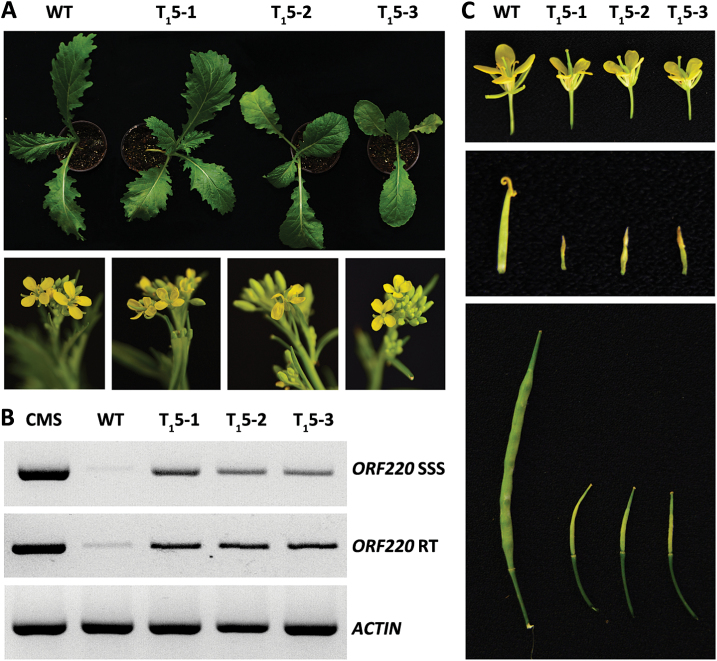
*MSH1-RNAi* construction in *B. juncea* and SSS of *ORF220*. (A) Seedling and inflorescence of WT and *MSH1-RNAi* lines. (B) SSS and transcript levels of *ORF220* in WT and *MSH1-RNAi* lines. (C) Flowers, stamens, and siliques of self-crossing from WT and *MSH1-RNAi* lines. (This figure is available in color at *JXB* online).

Two *MSH1* genes were isolated from the *B. juncea* genome with high amino acid sequence similarity to their ortholog in Arabidopsis (see Supplementary Fig. S5 at *JXB* online). We developed four independent *MSH1-RNAi* lines of *B. juncea* with confirmed suppression of *MSH1* expression, where two of the lines showed male sterility in the T_1_ generation. Varied leaf shape and normal flowering were also observed in the *MSH1-RNAi* lines (Fig. 5A). *ORF220* copy number assays showed evidence of SSS following *MSH1* suppression (Fig. 5B), and transcript levels of *ORF220* were correspondingly increased in *MSH1-RNAi* lines (Fig. 5B). Comparison of three other mitochondrial genes in these lines indicated no evidence of gene alteration or copy number shifting (Supplementary Fig. S6). *B. juncea MSH1-RNAi* lines produced small flowers (Fig. 5C), and stamen development was severely affected, such that anthers were not observed (Fig. 5C). The three *MSH1-RNAi* lines produced no seed by self-pollination (Fig. 5C), although seed set occurred with pollen from the WT. These results indicate that *MSH1* suppression can lead to SSS of *ORF220* and male sterility in *B. juncea*.

### Anther development-associated gene expression

We employed RNA-seq to identify global transcriptional differences between CMS and REV19 lines of *B*. *juncea*, and to investigate the nature of mitochondrial retrograde regulation associated with fertility reversion. In total, we found 4880 differentially expressed genes between CMS and REV19 lines (see Supplementary Table S4; Supplementary Fig. S7). The identified genes were involved in metabolic processes, response to stimulus, biological regulation, developmental processes, reproduction, and reproductive processes by GO analysis (Supplementary Fig. S8). By COG analysis, the differentially expressed genes involved functions in replication, recombination and repair, energy production and conversion, carbohydrate transport and metabolism, cell cycle control, cell division, chromosome partitioning, and signal transduction (Supplementary Fig. S9).

**Fig. 6.**
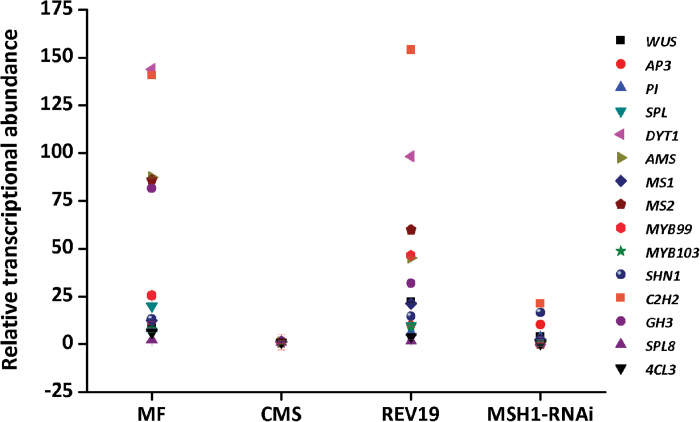
Transcriptional analysis of anther development-associated genes in fertile maintainer, CMS, REV19, and *MSH1-RNAi* lines of *B. juncea*. (This figure is available in color at *JXB* online).

We selected 15 anther development-associated genes (Table 1) to investigate transcription patterns among MF, CMS, REV19, and *MSH1-RNAi* lines of *B*. *juncea*. Expression of these anther development genes is up-regulated in REV19 compared with CMS lines, accompanied by reversion from male-sterile to fertile. Moreover, these genes are down-regulated in the *MSH1-RNAi* line compared with WT, with transition from male fertile to sterile (Fig. 6). For example, at early-stage initiation of anther development, the expression of *WUS* and several MADS-box genes, including *AP3*, *AG*, and *PI*, increased in REV19 relative to CMS, and showed decrease in the *MSH1-RNAi* line relative to WT (Fig. 6). During anther morphogenesis, the key regulatory gene for microsporogenesis *SPOROCYTELESS* (*SPL*) was restored to normal transcript levels in REV19, and decreased in *MSH1-RNAi* compared with WT (Fig. 6). We also found that expression of *DYT1*, *AMS*, *MS1*, *MS2*, *MYB99*, and *MYB103* were correspondingly increased in REV19 compared with CMS, and decreased in the *MSH1-RNAi* line compared with WT at late-stage tapetum function and pollen development (Fig. 6). These results indicate that male-sterility induction by MSH1 suppression and fertility reversion—via SSS—are accompanied by corresponding changes in anther-associated gene expression, implying a relationship between mitochondrial genome behavior and anther development programs.

**Table 1.** Transcriptional analysis of anther development-associated genes by RNA-seq

Gene ID	CMS reads	REV19 reads	Log_2_ FC (CMS/REV19)	Ortholog in Arabidopsis	Annotation
*Bju009726*	4	42	–3.325892244	*WUS*	*WUSCHEL*, homeobox gene controlling the stem cell
*Bju047574*	108	321	–1.881915185	*AP3*	*APETELA3*, floral homeotic gene encoding a MADS domain transcription factor
*Bju012907*	138	308	–1.472805418	*PI*	*PISTILLATA*, floral homeotic gene encoding a MADS domain transcription factor
*Bju010658*	1	35	–4.246938286	*SPL*	*SPOROCYTELESS*, initiation of micro- and megagametogenesis
*Bju083268*	22	855	–5.518191891	*DYT1*	DYSFUNCTIONAL TAPETUM 1
*Bju076135*	12	634	–5.892604764	*AMS*	ABORTED MICROSPORES
*Bju004296*	0.01	34	–4.992803589	*MS1*	MALE STERILITY 1
*Bju014803*	43	1911	–5.752855182	*MS2*	MALE STERILITY 2
*Bju003047*	1	178	–6.557555136	*MYB99*	MYB transcription factor
*Bju027475*	0.01	21	–4.325437835	*MYB103*	MYB transcription factor
*Bju072885*	2	47	–4.154850764	*At1g02040*	zinc finger (C2H2 type) family protein
*Bju*024267	56	169	–1.891531152	*4CL3*	pollen exine formation
*Bju*028126	32	2446	–4.325437835	*AT5G13380*	Auxin-responsive GH3 family protein, pollen exine formation
*Bju*002493	54	186	–2.080138807	*SHN1/WIN1*	ERF/AP2 transcription factor
*Bju038196*	109	26	1.702239773	*SPL8*	SQUAMOSA PROMOTER BINDING PROTEIN-LIKE 8

## Discussion

**Fig. 7.**
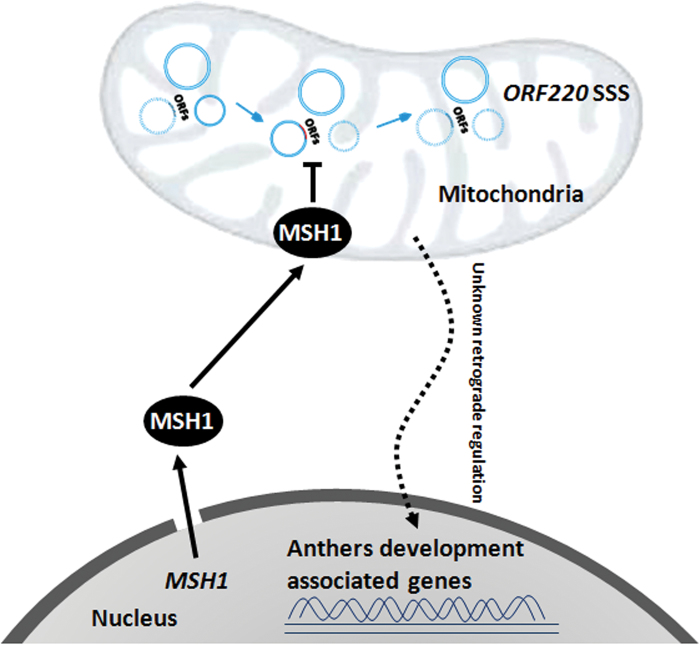
A working model for MSH1 mediating ORF220 SSS and causing male sterility. (This figure is available in color at *JXB* online).

CMS and fertility restoration are valuable components of hybrid breeding systems in crops, deriving from competitive interactions between mitochondrial and nuclear genomes (Ma, 2013; Chen and Liu, 2014). Spontaneous fertility reversion sporadically occurs in some CMS systems, providing insight into the relationship of mitochondrial SSS and plant reproductive behavior (Escote *et al.*, 1985; Rottmann *et al.*, 1987; Janska *et al.*, 1998; Feng *et al.*, 2009). These spontaneous fertility reversion events are influenced in frequency by nuclear background (Mackenzie *et al.*, 1988; Small *et al.*, 1988), and can be problematic to commercial interests for CMS implementation. In most fertility reversion cases previously reported, the nuclear genes involved in triggering mitochondrial genome rearrangement are largely unknown. In the case of *MSH1*, previous evidence suggests that the loss of *MSH1* function creates conditions conducive to mitochondrial asymmetric DNA exchange (Davila *et al.*, 2011). We propose that the spontaneous SSS of *ORF220* for fertility reversion in *B. juncea* is associated with processes controlled, at least in part, by *MSH1* (Fig. 7).

### SSS of ORF220 is associated with spontaneous fertility reversion

Mitochondrial DNA rearrangements are often observed in some CMS systems upon reversion to fertility (Fauron *et al.*, 1987; Smith *et al.*, 1987; Escotecarlson *et al.*, 1988; Mackenzie *et al.*, 1988; Bellaoui *et al.*, 1998; Janska *et al.*, 1998), with dramatic reduction in relative copy number of the CMS sequence in each case. Here, we demonstrated that the SSS of CMS-associated *ORF220* occurs in association with fertility reversion in CMS *B. juncea*. To confirm that ORF220 is sufficient to condition the CMS phenotype, *ORF220* was mitochondrially targeted, and the transgenic plants displayed male sterility in both Arabidopsis and *B. juncea* (Yang *et al.*, 2010). The amenability of this system to both transgenic induction and to fertility reversion provides a valuable opportunity for more detailed investigations of factors influencing nuclear–mitochondrial stability.

### Depressed expression of *MSH1* caused SSS of *ORF220* and male sterility

It is not clear the extent to which *MSH1* variation might have influenced spontaneous CMS reversion in natural systems. In the case of CMS common bean, SSS of the CMS-associated *pvs-orf239* was associated with changes in a single nuclear gene that, at that time, was designated *Fr* (Mackenzie and Chase, 1990). It has not been determined whether *Fr* might represent *MSH1* or a gene modulating *MSH1*.

Plant mitochondrial genome stability is controlled by nuclear recombination surveillance mechanisms that include at least three nuclear genes, *MSH1*, *RecA3*, and *OSB1* (Zaegel *et al.*, 2006; Shedge *et al.*, 2007; Arrieta-Montiel *et al.*, 2009). Of these three genes, disruption of *MSH1* can also influence male sterility (Sandhu *et al.*, 2007). Here, we present evidence that further substantiates this causality by SSS of CMS-associated *ORF220* when *MSH1* is suppressed. Pollen fertility was significantly reduced in *MSH1-RNAi* lines of *B. juncea*. However, the *MSH1*-associated SSS process observed in Arabidopsis Col-0 does not result in male sterility, indicating that not all SSS events necessarily give rise to a CMS phenotype (Abdelnoor *et al.*, 2003). This response in Arabidopsis may be due to the lack of a CMS-associated mitochondrial sequence in the Col-0 ecotype (Gobron *et al.*, 2013). Furthermore, only partial male sterility was observed in *MSH1-RNAi* lines of *B. juncea* in this study, and in *MSH1-RNAi* lines of tomato and tobacco plants previously (Sandhu *et al.*, 2007). SSS events are bidirectional and dynamic, so that only a fraction of CMS-associated genes might achieve a threshold to cause male sterility.

This fertility reversion mechanism is distinguished from fertility recovery by nuclear fertility restoration. CMS-associated gene expression has been observed to be modulated by nuclear restorer genes, which also sometimes affect mitochondrial metabolism to confer biochemical activities that facilitate pollen development (Liu *et al.*, 2001; Hanson and Bentolila, 2004; Chase, 2007; Ma, 2013; Chen and Liu, 2014). In nature, it is reasonable to assume that both restorer and reversion mechanisms are operational, with restorer systems providing a means of recovering self-fertility under conditions when CMS plants are located within cross-compatible populations, and reversion providing a means of self-pollination when CMS plants are in reproductive isolation.

### Fertility reversion is associated with anther development-associated gene expression

Early anther development includes stamen identity determination, lobed anther structure morphogenesis, anther cell layer specification, and early microspore development processes. Molecular genetic studies have uncovered crucial molecules and transcription factors that function in determining anther cell types and in controlling gene expression regulatory networks for anther development (Chang *et al.*, 2011a; Pearce *et al.*, 2015). We observed that the reproductive dynamics created by manipulating mitochondrial genome behavior in *B. juncea* includes altered expression of several anther development genes in CMS, REV19, WT, and *MSH1-RNAi* lines. Increased expression of *WUSCHEL* (*WUS*), *APETELA3* (*AP3*), and *PISTILLATA* (*PI*) occurred with recovery of floral structure development in the REV19 line. Correspondingly, reduced expression of these genes may cause the abnormal adhesive structure of petal and stamen observed in *MSH1-RNAi* lines. *SPOROCYTELESS* (*SPL*)—essential for the formation of reproductive cells and microsporogenesis—was altered in expression, suggesting its role in specifying the reproductive cell fate in these lines (Liu *et al.*, 2009). We observed increased expression of *SPL* in REV19 and decreased expression in *MSH1-RNAi* lines, suggesting the action of *SPL* in fertility conversion. These results indicate that male sterility and fertility reversion, caused by the SSS of *ORF220* and mediated by MSH1, involve differential regulation of anther development networks.

A distinctive characteristic of plant mitochondrial genomes is their recombinational versatility. The SSS activity of mitochondrial genomes likely serves as an important mechanism for maintaining appropriate function while retaining mitochondrial adaptive genetic diversity (Small *et al.*, 1987). Our findings here suggest that it is feasible to directly manipulate the *MSH1*-mediated sterility–fertility reversion mechanism in crops, a promising first step toward enhancing breeding potential by creating CMS or controlling fertility reversion behavior.

## Supplementary data

Supplementary data are available at *JXB* online.

**Supplementary Fig. S1.** Candidate revertant lines of *B. juncea*.

**Supplementary Fig. S2.** Comparison of mitochondrial DNA from CMS and revertant lines of *B. juncea*.

**Supplementary Fig. S3.** Mitochondrial genome rearrangement of the *atpA* gene.

**Supplementary Fig. S4.** Schematic diagram of *ORF220* gene construction.

**Supplementary Fig. S5.** *MSH1* from *B. juncea* and comparison with its ortholog in *Arabidopsis thaliana*.

**Supplementary Fig. S6.** SSS of mitochondrial genes in *MSH1-RNAi* lines relative to WT.

**Supplementary Fig. S7.** Genes differentially expressed between CMS and REV19 lines of *B. juncea* by RNA-seq.

**Supplementary Fig. S8.** Gene Ontology enrichment analysis of differentially expressed genes.

**Supplementary Fig. S9.** COG analysis of differentially expressed genes.

**Supplementary Table S1.** Candidate revertant events from CMS *B. juncea*.

**Supplementary Table S2.** Fertility of ORF220 expression in Arabidopsis.

**Supplementary Table S3.** Primers used in this study.

**Supplementary Table S4.** Genes differentially expressed between CMS and REV19 by RNA-seq.

**Supplementary data.** Assembled mitochondrial genomic scaffolds of CMS and REV19 lines.

Supplementary Data
