# Supplementary material for: MutS HOMOLOG1 silencing mediates ORF220 substoichiometric shifting and causes male sterility in Brassica juncea
Source: J Exp Bot. 2015 Oct 29;67(1):435–44. doi: 10.1093/jxb/erv480 (PMC4682445; doi:10.1093/jxb/erv480)
Supplement: Supplementary Data [file supp_67_1_435__index.html]

MutS HOMOLOG1 silencing mediates ORF220 substoichiometric shifting and causes male sterility in Brassica juncea — MutS HOMOLOG1 silencing mediates ORF220 substoichiometric shifting and causes male sterility in Brassica juncea — Supplementary Data 

# MutS HOMOLOG1 silencing mediates *ORF220* substoichiometric shifting and causes male sterility in *Brassica juncea*

## Supplementary Data

Data files

- supplementary\_Data.Assembled\_mitochondrial\_genomic\_scaffolds\_of\_CMS\_and\_REV19\_lines.txt - Supplementary Data
- supplementary\_Figures\_S1\_S9\_Tables\_S1\_S3.pdf - Supplementary Data
- supplementary\_Table\_S4.xlsx - Supplementary Data
