# Supplementary material for: MutS HOMOLOG1 silencing mediates ORF220 substoichiometric shifting and causes male sterility in Brassica juncea
Source: J Exp Bot. 2015 Oct 29;67(1):435–44. doi: 10.1093/jxb/erv480 (PMC4682445; doi:10.1093/jxb/erv480)
Supplement: Supplementary Data [file supp_erv480_supplementary_Figures_S1_S9_Tables_S1_S3.pdf]

MutS HOMOLOG1 silencing mediates *ORF220* substoichiometric shifting and causes male sterility in *Brassica juncea*

Na Zhao, Xinyue Xu, Yashitola Wamboldt, Sally A. Mackenzie, Xiaodong Yang, Zhongyuan Hu, Jinghua Yang*, Mingfang Zhang

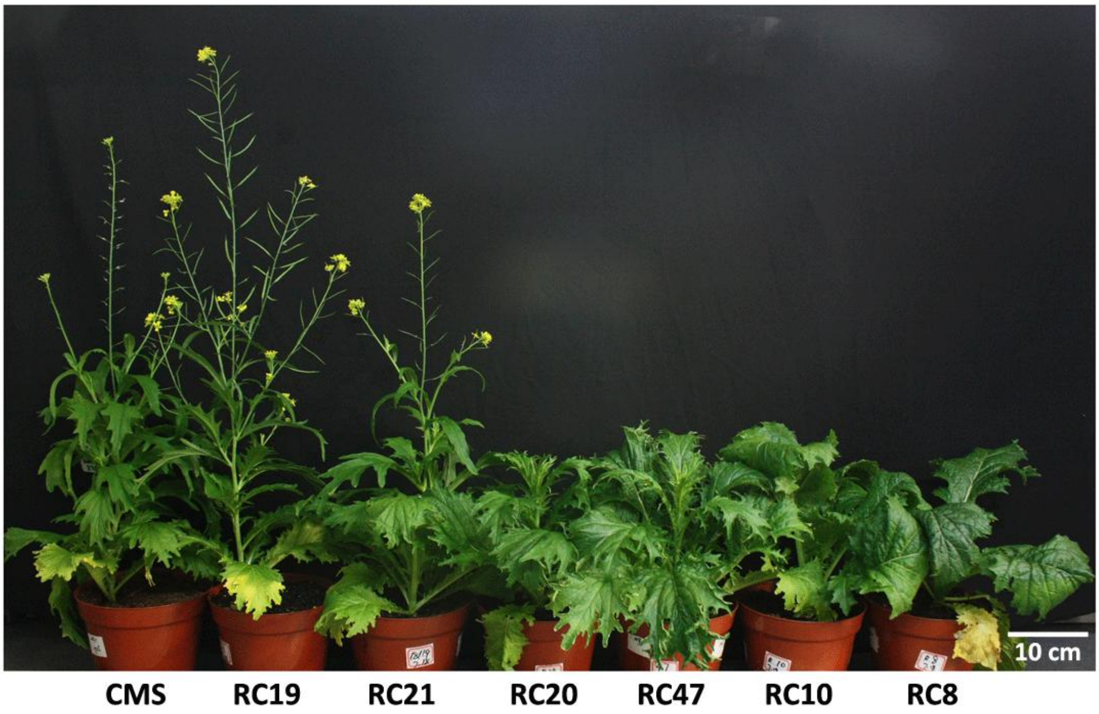

Figure S1 Candidate revertant lines of *B. juncea*.

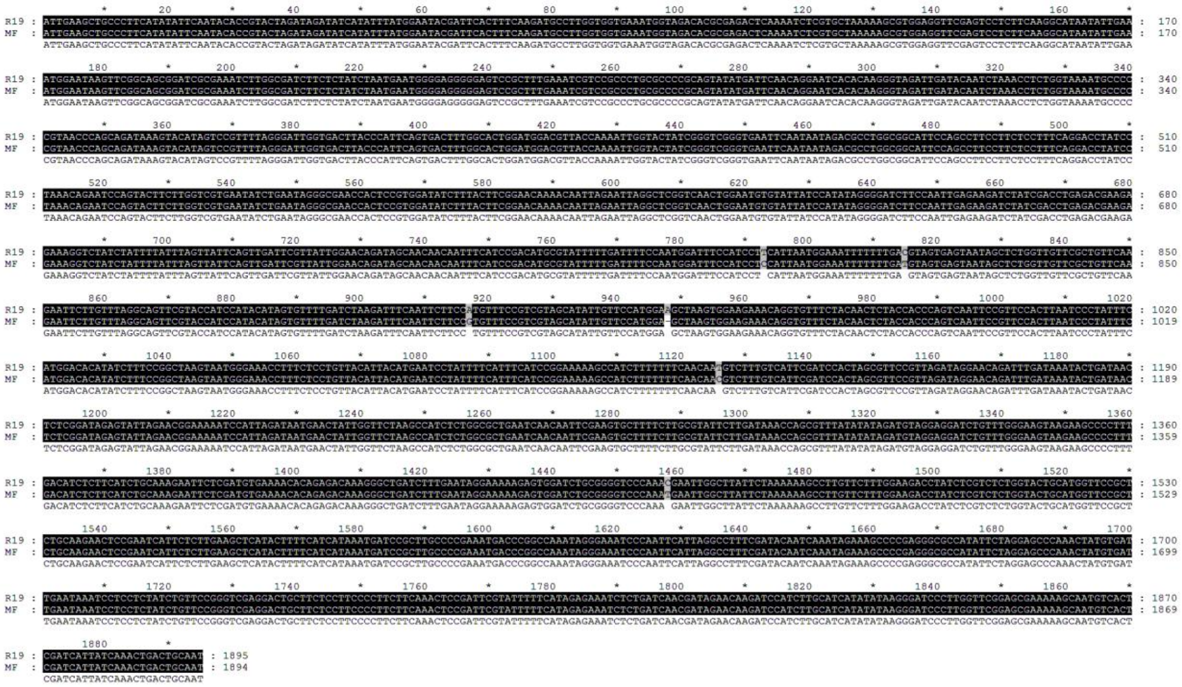

Figure S2 Comparison of mitochondrial DNA from CMS and revertant lines of *B. juncea*.

MF CMS R8 R10 R19 R20 R21 R47

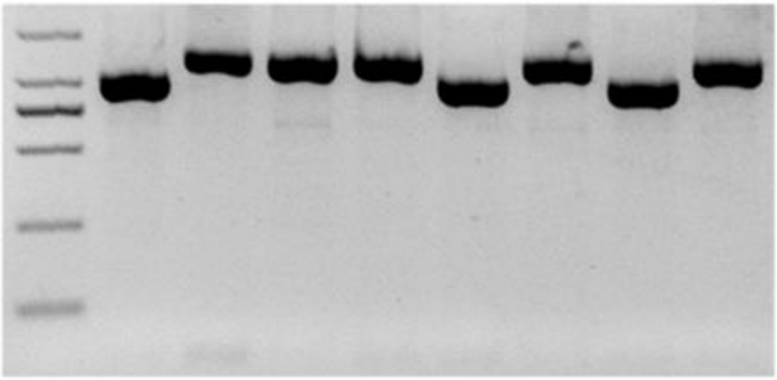

Figure S3 Mitochondrial genome rearrangement of the *atpA* gene.

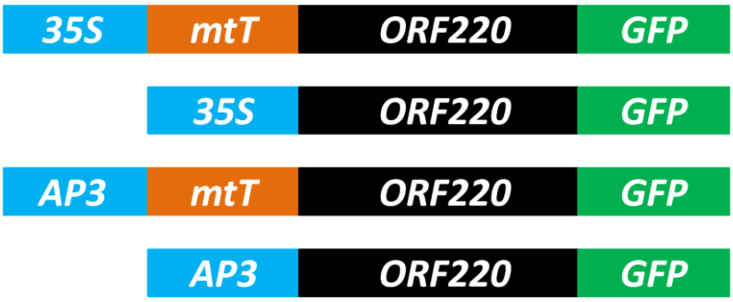

Figure S4 Schematic diagram of ORF220 gene construction.

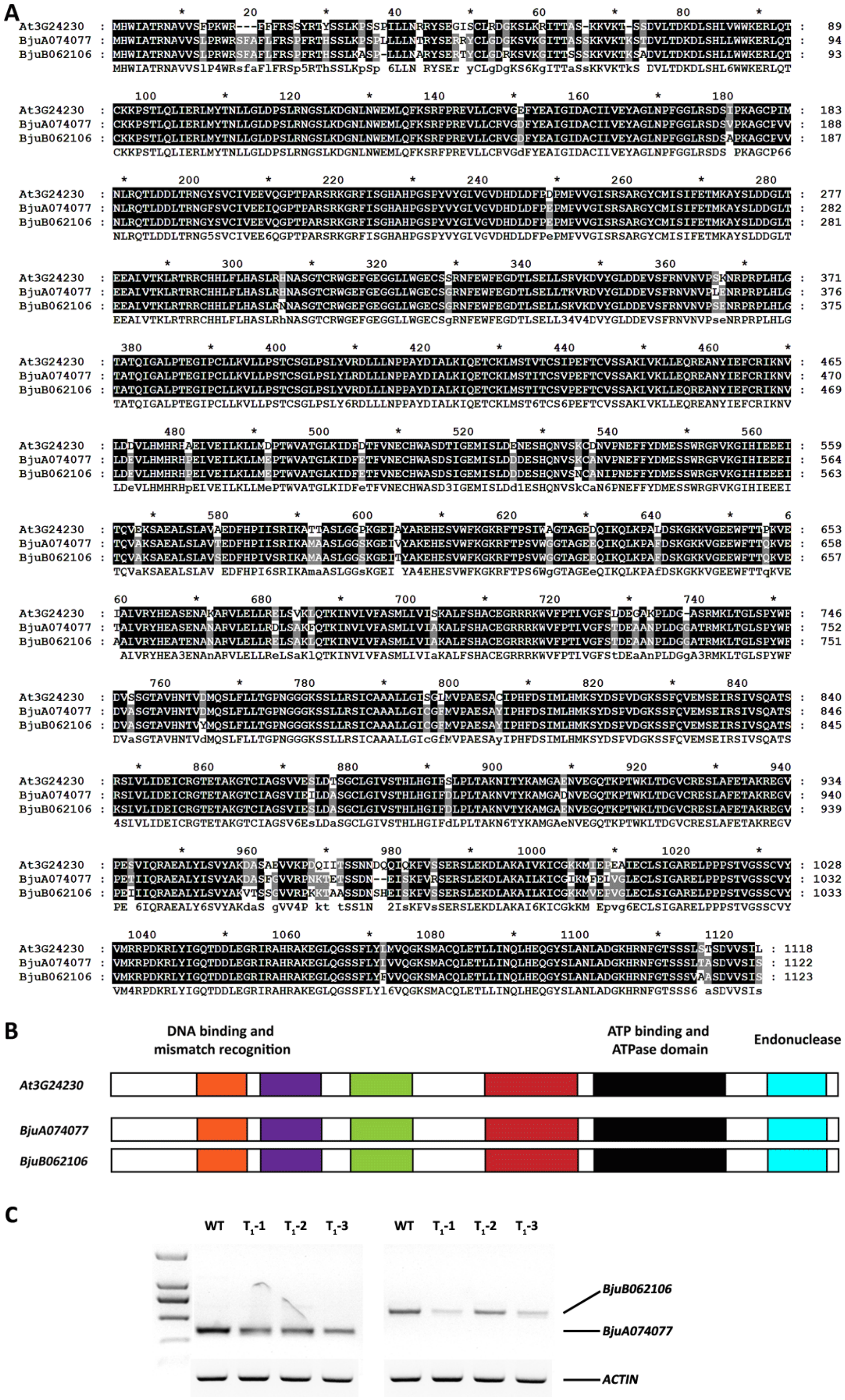

Figure S5 *MSH1* from *B. juncea* and comparison with its ortholog in *A. thaliana*.

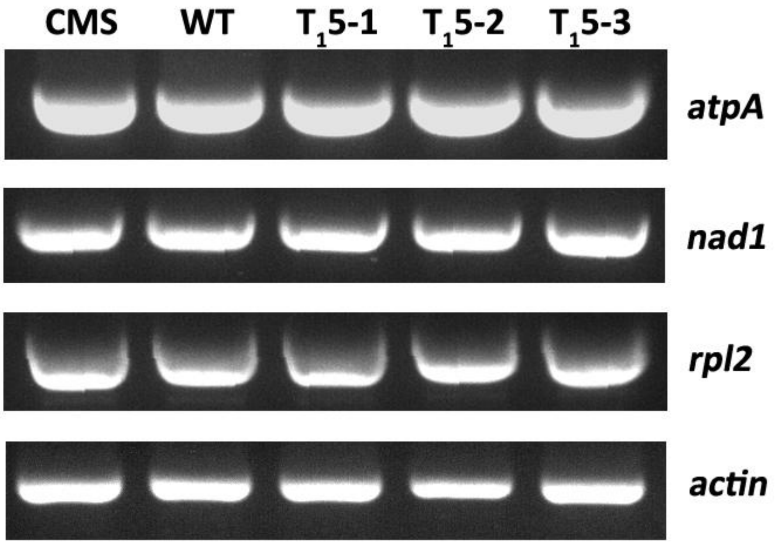

Figure S6 Substoichiometric shifting of mitochondrial genes in MSH1-RNAi lines relative to wild type.

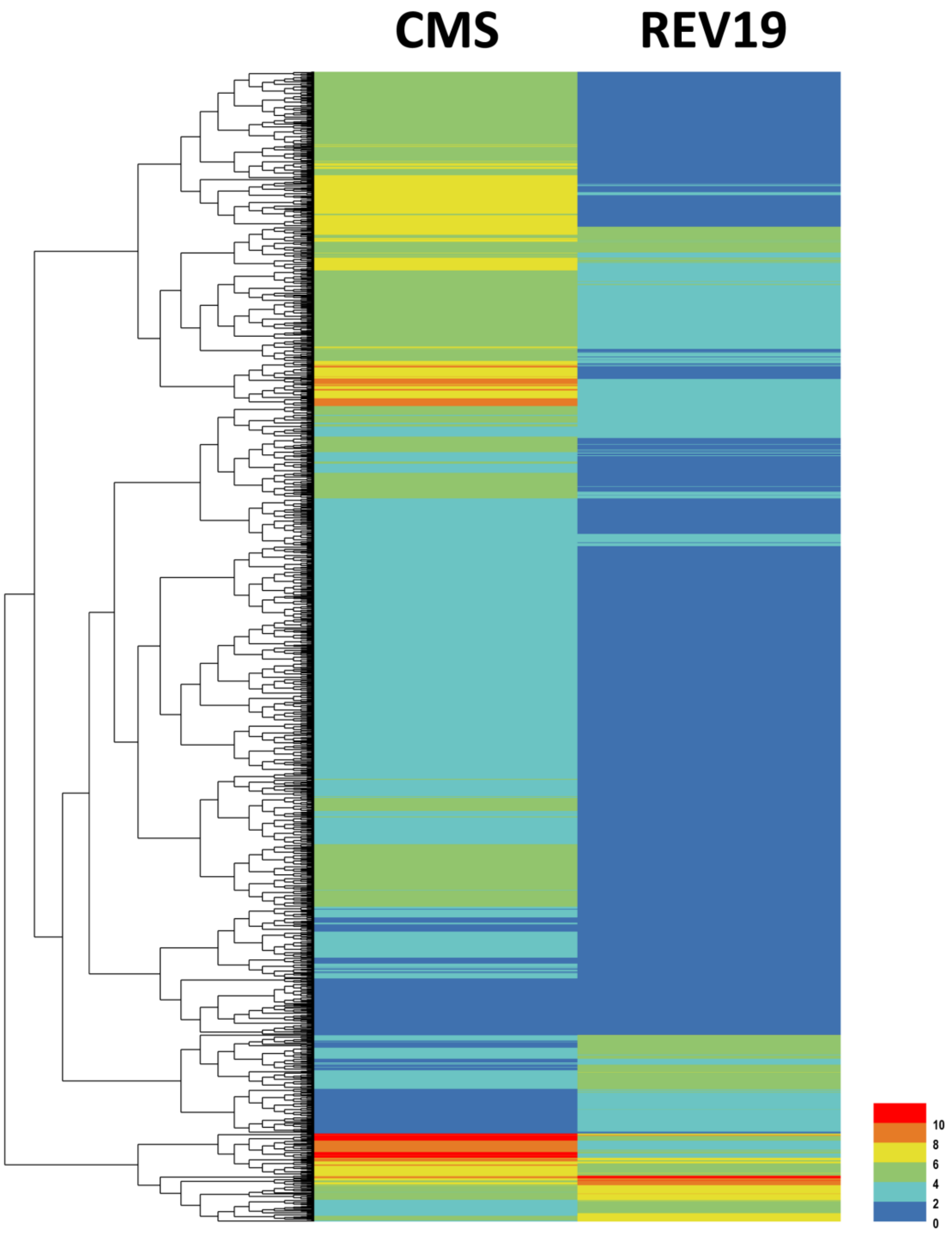

Figure S7 Genes differentially expressed between CMS and REV19 lines of *B. juncea* by RNA-seq.

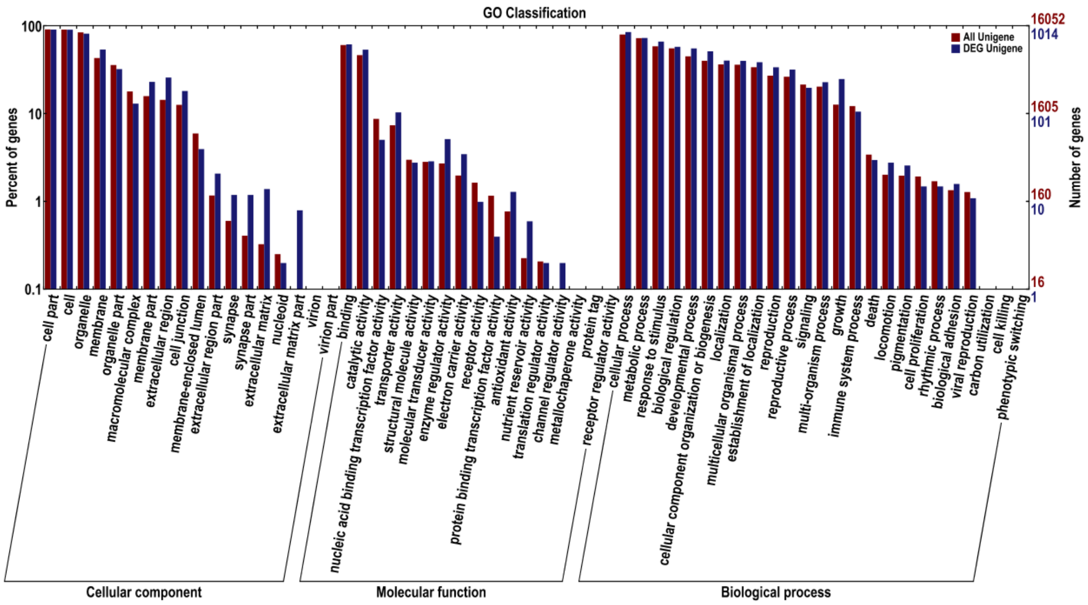

Figure S8 Gene Ontology (GO) enrichment analysis of differentially expressed genes.

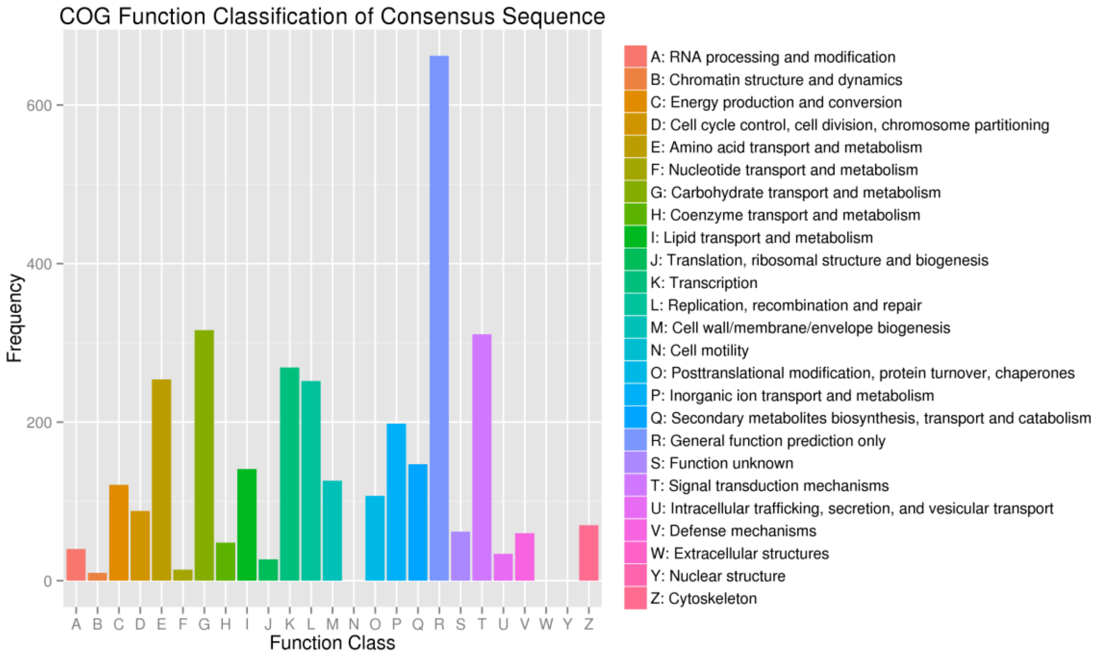

Figure S9 Clusters of Orthologous Groups (COG) analysis of differentially expressed genes.

Table S1 Primers used in this study

Gene	Forward primer	Reverse primer
ORF220-SSS	ATGCCTCAACTGGATAAATTCACTT	TCATCGATAGAGATCGAGAATTTCG
atpA	GCTGCTTACAGGAGTTAGCC	GTCCAATCGCTACATAGACA
ACTIN	CGCCGAGCGGGAAATCGTC	GGAAAGTGCTGAGGGATGC
ORF220-Construction	CGAAACCAACTGCTTTCACA	TTCGTTCATCCTGGCTCTCT
AP3 promoter	CAGTAACTGTGGCCAACTTAGT	ATTCTTCTCTCTTTGTTTAATCTTT
mt-targeted	CCATGGCTTCTCGGAGGCTTCT	CGCTGCGGAGGTAGCGTACTG
RT-MSH1-A	AAGAATACGTGCGCATAGGG	AAGCAAAAACGGCATACATTG
RT-MSH1-B	GCTTCATCGGACAATAGCCAC	CCTGCCGTTTGCAATATCTC
Q-ORF220	GGAGATTCGAAGCCTACAGC	ATCGAGGATCTCGTTCATCC
Q-AMS	TACCGGGAGGATTAGTGGAG	TCATGTTAATCGTTGCGGTT
Q-AP3	GGTGTTGTGTGATGCTAGGG	CAACATCAGAAACCGTTTGG
Q-C2H2	ACAACACGATGAGGGCAATA	AAGCATTCCCTGAATTGGAC
Q-DYT1	CATGGGAAGTGAACTTGTGG	TTTAGGTATCGGTCCAAGCC
Q-GH3	AAGCGCAACACATTTACTCG	ACTTTCGCGTCCTTGAGTTT
Q-MYB99	GAGGTTGGACTCAGAAAGGG	GCTTGCTCCACGTGTTCTTA
Q-MYB103	CACGGTGGAATACGTACAGC	CCACCACTTCTCCCTTCAAT
Q-MS1	CTCATGGGAAGTTGGTCCTT	GCAACGGAGGTTAAGAGAGG
Q-MS2	GCGGTTAAAGAACGAGGTGT	TCTGTTTGCAACCCAATGTT
Q-PI	GCTGCAACAACAAGAGATGG	GGTTGAATCGGCTGAACTCT
Q-SHN1	CTGACATCTCAGCCTCCTCA	AGACTTGCAGCATTTCCTCA
Q-SPL	GTACTTCAACGAGGCGACAA	GATGTAGCCACTGGAAACGA
Q-SPL8	GCTCGATTTCACTTCCAACA	TACAGCCTGCTCACGAAATC
Q-WUS	CCAAGAGAGCGGTAACAACA	AGGATTCTGATCTGCTCCGT
Q-4CL3	CACCGTTATCACCACAGAGG	GTGCCAGAGGAGAAAGGAAG
Q-25S	CGGTTCCTCTCGTACTAGGTTGA	CCGTCGTGAGACAGGTTAGTTTT

Table S2 Statistic of candidate revertant event from CMS *B. juncea*

CMS plant number	Seed number	Sterile	Fertile
1	5	5	0
2	5	5	0
3	4	4	0
4	4	4	0
5	3	3	0
6	3	3	0
7	3	3	0
8	2	2	0
9	2	2	0
10	2	2	0
11	2	2	0
12	2	2	0
13	1	1	0
14	1	1	0
15	1	1	0
16	1	1	0
17	1	1	0
18	1	1	0
19	7	5	2
20	1	1	0
21	1	1	0
22	1	1	0
From No. 23 - No.39	0	0	0
Total	53	51	2

Table S3 Statistic of fertility of ORF220 expression in *Arabidopsis*

	ORF220		*mt target-ORF220*	
	Sterile	Fertile	Sterile	Fertile
35S promoter	0	20	17	42
AP3 promoter	1	13	28	0
